# Oligonucleotide-Functionalized Gold Nanoparticles for Synchronous Telomerase Inhibition, Radiosensitization, and Delivery of Theranostic Radionuclides

**DOI:** 10.1021/acs.molpharmaceut.1c00442

**Published:** 2021-08-27

**Authors:** Bas M. Bavelaar, Lei Song, Mark R. Jackson, Sarah Able, Ole Tietz, Irini Skaripa-Koukelli, Philip A. Waghorn, Martin R. Gill, Robert C. Carlisle, Madalena Tarsounas, Katherine A. Vallis

**Affiliations:** † Oxford Institute for Radiation Oncology, University of Oxford, Old Road Campus Research Building, Roosevelt Drive, Oxford OX3 7DQ, U.K.; ‡ Institute of Cancer Sciences, Wolfson Wohl Cancer Research Centre, University of Glasgow, University Avenue, Glasgow G12 8QQ, U.K.; § Charles River Laboratories, Elphinstone Research Centre, Elphinstone, Tranent EH33 2NE, U.K.; ∥ Institute of Biomedical Engineering, Department of Engineering Science, University of Oxford, Old Road Campus Research Building, Oxford OX3 7DQ, U.K.

**Keywords:** telomerase, targeted radionuclide therapy, gold nanoparticles, Auger electrons, nanomedicine

## Abstract

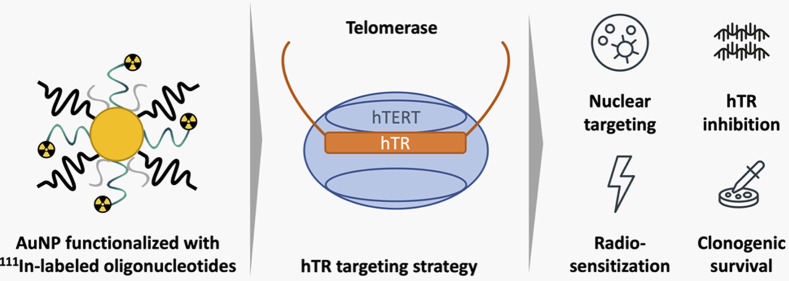

Telomerase represents an attractive target in oncology as it is expressed in cancer but not in normal tissues. The oligonucleotide inhibitors of telomerase represent a promising anticancer strategy, although poor cellular uptake can restrict their efficacy. In this study, gold nanoparticles (AuNPs) were used to enhance oligonucleotide uptake. “match” oligonucleotides complementary to the telomerase RNA template subunit (hTR) and “scramble” (control) oligonucleotides were conjugated to diethylenetriamine pentaacetate (DTPA) for ^111^In-labeling. AuNPs (15.5 nm) were decorated with a monofunctional layer of oligonucleotides (ON–AuNP) or a multifunctional layer of oligonucleotides, PEG(polethylene glycol)800-SH (to reduce AuNP aggregation) and the cell-penetrating peptide Tat (ON–AuNP–Tat). Match–AuNP enhanced the cellular uptake of radiolabeled oligonucleotides while retaining the ability to inhibit telomerase activity. The addition of Tat to AuNPs increased nuclear localization. ^111^In–Match–AuNP–Tat induced DNA double-strand breaks and caused a dose-dependent reduction in clonogenic survival of telomerase-positive cells but not telomerase-negative cells. hTR inhibition has been reported to sensitize cancer cells to ionizing radiation, and ^111^In–Match–AuNP–Tat therefore holds promise as a vector for delivery of radionuclides into cancer cells while simultaneously sensitizing them to the effects of the emitted radiation.

## Introduction

Telomerase is a ribonucleoprotein with a key role in the replicative immortality of cancer. Human telomerase is composed of a telomerase RNA subunit (hTR), which contains the internal template for the synthesis of telomeric hexanucleotide DNA repeats, and an enzymatic component, telomerase reverse transcriptase (hTERT), which catalyzes the addition of nucleotides at telomere ends. In addition, a number of accessory proteins (dyskerin, NHP2, NOP10, and GAR1) are associated with telomerase.^1,2^ The holoenzyme catalyzes *de novo* addition of TTAGGG nucleotide telomeric repeats to chromosome ends. Telomeres erode in somatic cells during each cell division as a result of the inability of the DNA synthesis machinery to perform complete replication (the “end replication problem”).^3^ Telomere shortening limits the number of possible cell divisions since critically short telomeres induce senescence or apoptosis.^4,5^ In 85% of cancers, however, telomerase activity is upregulated, counteracting the process of telomere shortening and conferring unlimited proliferative potential to malignant cells.^6^ As a result, therapeutic strategies have been developed to inhibit telomerase function in cancers.^7^

One of the most promising therapeutic approaches is the use of anti-hTR oligonucleotides that bind the RNA template and thus act as catalytic inhibitors of telomerase.^8−11^ Therapeutic efficacy has been demonstrated in various *in vitro* and *in vivo* settings, and positive results have been reported for Imetelstat/GRN163L in essential thrombocytopenia and myelofibrosis.^12,13^ Interestingly, several studies have shown that the use of oligonucleotide template inhibitors causes cancer cell sensitization to radiotherapy and chemotherapy as a result of telomere length-dependent and telomere length-independent mechanisms.^14−20^ To exploit this radiosensitizing effect, an oligonucleotide hTR inhibitor was conjugated to indium-111 (^111^In) for concomitant telomerase inhibition and targeted radionuclide therapy (TRT).^21^^111^In emits Auger electrons that cause dense ionizations over a short range (nm to μm), thus allowing for irradiation of individual cells and sparing of non-targeted tissue.^22,23^ This ^111^In-labeled hTR-targeted oligonucleotide construct causes sequence- and telomerase-dependent DNA damage and cell-killing effects in cancer cells.^21^ However, the use of oligonucleotides as anticancer agents is hampered by poor cellular uptake and unfavorable pharmacokinetics. There is therefore an impetus in the field of DNA therapeutics to devise new oligonucleotide delivery methods that can overcome this barrier to the clinic.^24^

Nanosized drug carriers provide a platform for target-specific delivery.^25^ In particular, gold nanoparticles (AuNPs) exploit unique physicochemical properties that can augment intra-cellular uptake of oligonucleotides. The AuNP surface can be decorated with multiple moieties, allowing the design of nanoparticles with a shell of targeting agents, cell-penetrating peptides (CPPs), oligonucleotides, and other elements.^25^ AuNP–oligonucleotide constructs have been used for various applications, including antisense gene control,^26^ gene expression knockdown with siRNA,^27^ intracellular detection of RNA,^28^ and TRT.^29−31^ However, their potential as a vector for hTR inhibition has not been explored, and the combination with Auger electron radiotherapy has not been tested previously.

Here, we describe the design, characterization, and *in vitro* effect of a dual-modality therapeutic approach that uses ^111^In-labeled anti-hTR oligonucleotide-functionalized AuNP constructs. Radiolabeled anti-hTR oligonucleotides were conjugated to 15.5 nm AuNPs to increase their cellular uptake and resistance to endonuclease degradation. AuNPs were further modified with attachment of polyethylene glycol (PEG) for stability and Tat, a CPP/nuclear localization sequence (NLS), for cellular and nuclear delivery.^32−35^ Detailed *in vitro* characterization indicates that ^111^In-labeled anti-hTR oligonucleotide- and Tat-functionalized AuNP constructs have a telomerase-dependent cell-killing effect, thereby providing a new avenue for the treatment of telomerase-positive cancers.

## Experimental Section

### Tissue Culture

The melanoma cell line MDA-MB-435 and the osteosarcoma cell line U20S were used for cell studies. The cell lines were obtained from the American Type Tissue Culture Collection. The cells were kept in Dulbecco’s modified Eagle’s medium (Sigma-Aldrich #D5796) with 10% fetal bovine serum (Gibco #10270) and 1% penicillin/streptomycin/glutamate (Sigma-Aldrich #G1146). The cells were regularly passaged using 0.05% trypsin–EDTA (Gibson #25300-054) and checked monthly for mycoplasma contamination (MycoAlert testing kit; Lonza#LT07).

### Gold Nanoparticles

AuNPs (15.5 nm) were synthesized as described previously.^26^ The size and distribution of AuNPs were determined by dynamic light scattering (DLS) with a Zetasizer instrument (Malvern Instruments). The samples were diluted to a concentration of 5 nM in Milli-Q water and transferred to disposable plastic cuvettes at room temperature. The samples were measured in triplicate, and the hydrodynamic radius was calculated using Stoke–Einstein’s relation for the Brownian motion of particles. Particle size and morphology were further analyzed using transmission electron microscopy (TEM). The samples were lyophilized for 24 h on TEM grids of 150 mesh (Agar Scientific AGG201N) and images acquired using a Fei Tecnai 12 TE microscope and imaged with a 16 megapixel Gatan OneView camera with CMOS sensors. Images were obtained using a 1 s exposure time and a pixel saturation of 5000–6000. Analysis was performed using ImageJ software.

### Fluorophore Labeling of Oligonucleotides

High-performance liquid chromatography-purified 2′OMeRNA with hTR complementary (Match) and random (Scramble) oligonucleotide sequences with 5′-amino linkers and 3′-thiols were purchased from Sigma-Aldrich. Oligonucleotides were conjugated to Cy3-monoreactive dye (GE Healthcare #PA23001) according to the manufacturer’s protocol. Using P4 (BioRad #150-4120) size exclusion chromatography (SEC), reaction products were separated into 50 μL phosphate-buffered saline (PBS) fractions. The fractions were analyzed by a spectrophotometer at wavelengths of 260 nm (oligonucleotides) and 550 nm (Cy3).

### Radiolabeling of Oligonucleotides

Oligonucleotides were dissolved in NaHCO_3_ buffer (0.1 M, pH 8.3) to a concentration of 100 μM. Diethylenetriamine pentaacetate (DTPA) (Sigma-Aldrich #284025-1G) was dissolved in DMSO and added to the oligonucleotide solution in 10-fold molar excess. The reaction was incubated for at least 120 min. The reaction products were separated by SEC with P4-Biogel into 50 μL of sodium citrate (0.1 M, pH 5.0; Sigma-Aldrich #71498) fractions. The concentration was determined using a spectrophotometer at a wavelength of 260 nm. Radiolabeling was performed in sodium citrate (0.1 M, pH 5.0) by incubating DTPA-labeled oligonucleotides with ^111^InCl_3_ (PerkinElmer NEZ304A000MC) at a specific activity of 0.8 MBq/μg. Radiolabeling efficiency was determined using a radio thin-layer chromatography (TLC) imaging scanner (Bioscan #AR-2000).

### ON–AuNP Conjugates

ON–AuNP and ON–AuNP–Tat conjugates were synthesized as described previously.^26^ For ON–AuNPs, oligonucleotides (final concentration 3 μM) were added to a 15 nM solution of 15.5 nm AuNPs. For ON–AuNP–Tat, PEG800-SH (Sigma-Aldrich #729108) (final concentration 45 μM) was dissolved in a 15 nM AuNP solution for 20 min prior to the addition of oligonucleotides (final concentration 1.5 μM). After a 10 min incubation with oligonucleotides, Tat (sequence: GRKKRRQRRRPQGYGCG; Cambridge Peptides; final concentration 1.5 μM) was added to the solution. Both ON–AuNP and ON–AuNP–Tat were subsequently incubated overnight. On day 2, Au-NP-containing suspensions were salt-aged for 8 h to achieve a final sodium chloride (NaCl; Sigma-Aldrich #S7653) concentration of 0.3 M and then shaken overnight. The functionalized AuNPs were purified by centrifugation (20,000*g*; 40 min; 4 °C, three times) (Eppendorf #5417R), after which the pellet was resuspended in Milli-Q water. New batches of AuNPs were prepared prior to each experiment, and the particle size and morphology were determined as described above. The AuNP constructs were reconstituted in sodium citrate (0.1 M, pH 5.0) prior to radiolabeling. Subsequently, ^111^InCl_3_ at the desired specific activity was added and incubated overnight. The next day, radiolabeling was confirmed with TLC, and AuNPs were purified by centrifugation (20,000*g*; 40 min). The number of oligonucleotides per particle was determined by ^111^In-labeling oligonucleotides with a known ^111^In/oligonucleotide ratio prior to AuNP functionalization. ON–AuNP and ON–AuNP–Tat were synthesized as described above, and radiolabeled functionalized particles were centrifuged and washed to remove unbound ^111^In. Subsequently, the concentration of the pellet was measured with a spectrophotometer, and ^111^In counts per minute (CPM) in the pellet were measured using a Wizard3” automatic gamma-counter (PerkinElmer 2480). The number of oligonucleotides per pellet was calculated by comparing the ^111^In CPM of the resuspended pellet with a known standard of ^111^In CPM per oligonucleotide. The stated concentration in an experiment refers to the concentration of oligonucleotides conjugated to the AuNP. The ON–AuNP concentration and quality were determined by UV–vis spectrometry and DLS as described above. A final ON concentration of 210 nM was determined in clonogenic assays to exert the maximum effect and was used in all other studies unless stated otherwise.

### Telomeric Repeat Amplification Protocol

Cells were harvested and lysed according to the TRAPeze XL Telomerase Detection kit (EMD-Millipore #S7707) protocol. A known concentration of inhibitor was added to the reaction mix, and the polymerase chain reaction (PCR) protocol was started with the following alterations: the first cycle was set to 30 °C for 30 min, followed by 36 cycles at 94 °C for 30 s, 53.5 °C for 30 s, 72 °C for 60 s, and a final extension at 72 °C for 3 min. After PCR, the samples were centrifuged (20,000*g*, 30 min, 4 °C) to remove AuNPs and measured using a plate reader.

### Internalization and Fractionation Assays

For internalization assays, the cells were incubated for 24 h, washed to remove membrane-bound AuNPs using 0.1 M glycine HCl at pH 2.5, and lysed with 0.1 M NaOH as previously described.^36^ A Nuclei EZ Prep Nuclei Isolation kit (Sigma-Aldrich #NUC101-1KT) was used to determine the subcellular distribution of AuNP constructs. The cytosol and nuclei were isolated according to the manufacturer’s protocol. After separation of fractions, the samples were counted in a Wizard3” automatic gamma-counter.

### Confocal Microscopy

The cells (2 × 10^5^/well) were seeded in eight-well chamber slides (Thermo Scientific #177402), incubated overnight, and then treated with 210 nM Cy3–Match–AuNP or Cy3–Match–AuNP–Tat for 2.5 or 24 h in 200 μL media. The cells were then washed in PBS before fixation in 200 μL of 4% formaldehyde (Sigma-Aldrich #252549) for 10 min at room temperature. Before the removal of the medium chamber from the slide, the cells were washed three times with PBS. A drop of Vectashield (Vector #H-1200) with 4′,6-diamidino-2-phenylindole (DAPI) was added to each well, after which the samples were mounted using a 24 × 24 mm coverslip (VWR International #6310127). Images were obtained using a laser scanning Leica microscope (TCS SP8). Confocal planes were imaged using line-scanned consecutive acquisition channels with a 63×/1.40 oil immersion lens (pixel dwell time, 0.64 μs; pin-hole, 1 Airy unit).

### Transmission Electron Microscopy

MDA-MB-435 cells were plated onto Thermanox plastic coverslips (13 mm diameter; Thermo Scientific, #174950) in six-well plates at a seeding density of 8 × 10^5^ cells and left to adhere overnight. Fresh medium was added before incubation with 210 nM Match–AuNP or Match–AuNP–Tat. After 24 h, the medium was removed, and cells were washed twice in 1,4-piperazinediethanesulfonic acid buffer (PIPES buffer; 0.1 M; pH 7.2; Sigma-Aldrich # P8203) for 5 min. The samples were prepared and stained as previously described.^29^ After staining, the sections were analyzed on a Tecnai 12 TEM and imaged with a 16 megapixel Gatan OneView camera with CMOS sensors. Images were obtained using a 1 s exposure time, and a pixel saturation of 5000–6000.

### Clonogenic Assays

The cells were seeded in 96-well plates at 2 × 10^4^ cells/well overnight. The cells were incubated with radiolabeled and non-radiolabeled free oligonucleotides, ON–AuNP and ON–AuNP–Tat (27 MBq/nmol), for 24 h. For radiosensitivity experiments, the cells were incubated with AuNP constructs for 24 h and then exposed to external ionizing radiation (IR) using a cesium-137 (^137^Cs)-irradiator (0.662 MeV) to deliver a dose of 2, 4, or 6 Gy. After incubation for 1 h, the cells were harvested following washing in PBS and 50 μL of trypsin–EDTA was added. The harvested cells were counted before plating in six-well plates at a density sufficient to give more than 75 colonies per sample. The untreated cells were typically seeded at 600 cells/well. Colonies were grown for at least 7 days, washed in PBS, and stained with 1% methylene blue (Alfa Aesar #A18174) in 50% methanol (Fisher Scientific #M/4000/PC17). Excess stain was washed off with water. Colonies containing more than 50 cells were counted. The surviving fraction (SF) was calculated using the plating efficiency of untreated cells. The mean inactivation dose (MID), defined as MID = ∫_0_^∞^SF(*D*)d*D*, was calculated for each curve following fitting of a linear-quadratic model.^37,38^ MIDs were compared using one-way ANOVA with Bonferroni correction for multiple comparison. For radiosensitivity assays, the sensitizer enhancement ratio (SER) was calculated by dividing the MID of the non-AuNP-treated sample by the MID of the AuNP-treated sample (SER = MID_No AuNP_/MID_AuNP_).

### γH2AX Assay

The cells (2 × 10^5^/well) were seeded in an eight-well chamber slide (Thermo Scientific #177402) and incubated overnight. The cells were treated with 210 nM ^111^In-labeled ON–AuNP–Tat (18 MBq/nmol) or medium for 24 h in 200 μL medium. The positive control consisted of cells exposed to external beam irradiation (4 Gy). After incubation, the cells were washed twice in PBS, fixed in paraformaldehyde, permeabilized with 1% Triton X-100 in PBS, and blocked for 1 h at 37 °C with 2% BSA in PBS.^39^ Fixed cells were incubated with the anti-γH2AX primary antibody (JBW301; Millipore; 1:800 dilution) for 1 h at 37 °C, washed three times, and exposed to AF488-labeled goat-anti-mouse antibody (Invitrogen; 1:250 dilution) for 1 h at 37 °C. The cells were washed three times with PBS before removal of the medium chamber. One drop of DAPI was added to each well, after which the samples were mounted with a 24 × 24 mm coverslip (VWR International #6310127). Images were obtained using a laser scanning Leica confocal microscope (TCS SP8). Confocal planes were imaged using line-scanned consecutive acquisition channels through a 63×/1.40 oil immersion lens. The number of γH2AX foci and nuclear surface area (NSA) were automatically calculated using ImageJ software. All data is expressed as foci/NSA.

### *In Vivo* Imaging and Biodistribution

^111^In–Match–AuNP–Tat or ^111^In–Scramble–AuNP–Tat (2.5 μg oligonucleotide; 10 MBq) was administered intravenously (i.v.) to female athymic nude mice bearing MDA-MB-435 xenografts on the right flank (*n* = 3 per group). Single-photon emission computerized tomography (SPECT) images were acquired 24, 48, and 72 h post-injection (p.i.) using the VECTor^4^CT system (Milabs). Whole-body images were acquired over 30 min per mouse with an ultrahigh-resolution rat/mouse 1.8 mm collimator (HE-UHR-RM) (6 timeframes, 30 s per bed position, 10 positions). A CT scan (55 kV, 0.19 mA) was performed for anatomical reference. SPECT images were reconstructed using MiLabs ImageJ software. Animals were euthanized after the 72 h p.i. imaging session, and organs were removed and transferred to pre-weighed tubes. Tubes were weighed again, and radioactivity was measured using a Wizard3” automatic gamma-counter. Measurements were decay-corrected to the activity at the time of injection. A calibration curve was used to convert CPM to MBq, allowing calculation of the percentage of injected dose per gram of tissue (% ID/g).

## Results

### Synthesis of hTR-Targeting AuNP Radiopharmaceuticals

**Figure 1:**
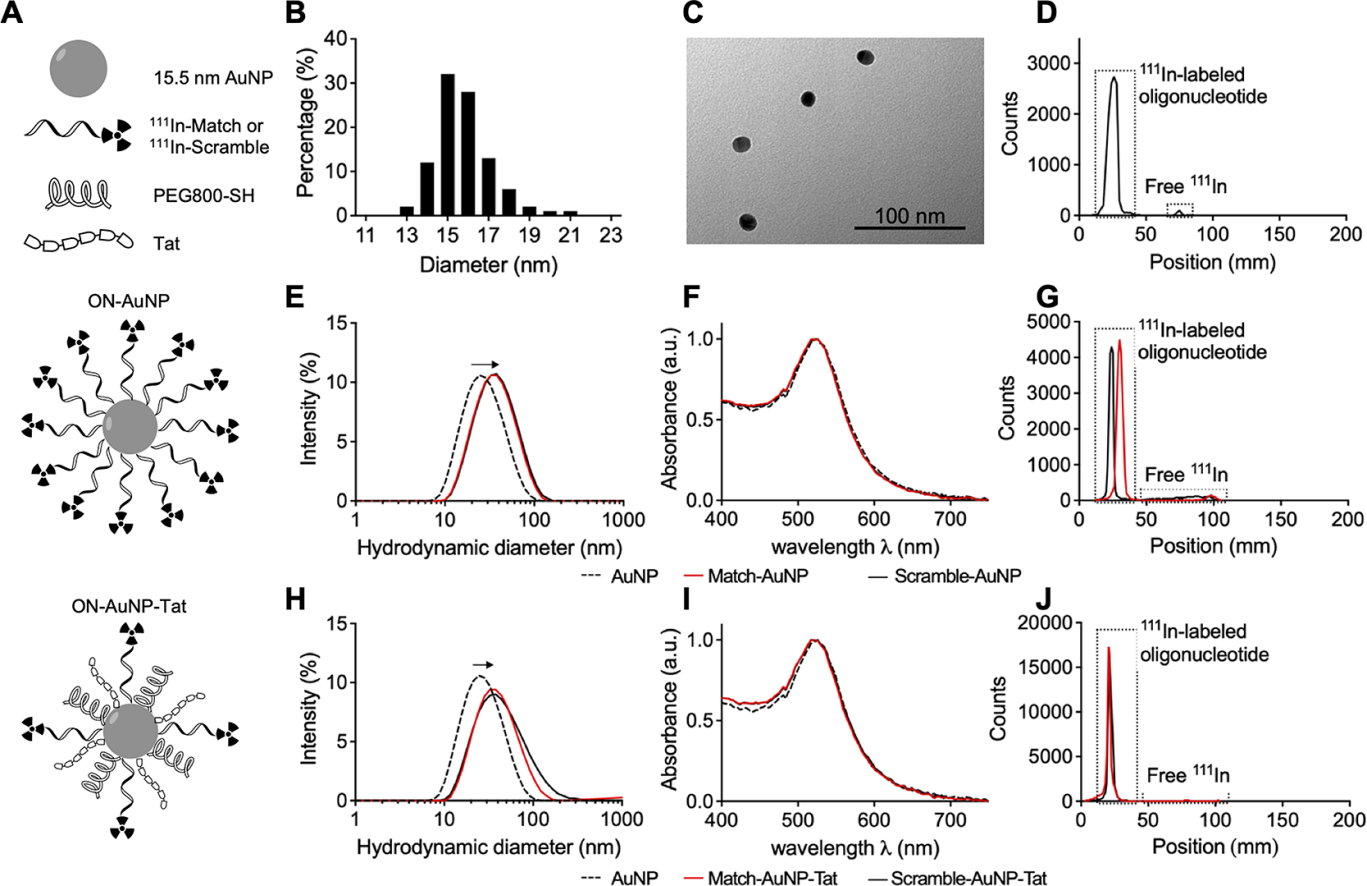
AuNP properties and functionalization. (A) Schematic of ^111^In–ON–AuNP and ^111^In–ON–AuNP–Tat. (B) Size distribution of 15.5 ± 1.9 nm AuNPs. (C) TEM image of AuNPs. (D) TLC reading of ^111^In–DTPA-labeled Match oligonucleotides. A peak at position = 25 mm, revealing the presence of ^111^In–Match, covers the surface of 98% of the graph. A small amount of radioactivity is seen at approximately 75 mm, indicating a trace amount of free ^111^In. (E) DLS of Match–AuNP and Scramble–AuNP in water. A rightward shift (from 25.4 to 33.3 nm) of Match–AuNP and Scramble–AuNP indicates surface functionalization. (F) UV–vis spectrum of ON–AuNP in water. The overlapping of the curves for AuNPs, Match–AuNP, and Scramble–AuNP suggests no aggregation, a rightward shift would indicate an increase in particle size. (G) Typical TLC result for ^111^In–Match–AuNP and ^111^In–Scramble–AuNP (radiochemical purity 95 and 90%, respectively). (H) DLS of Match–AuNP–Tat and Scramble–AuNP–Tat in water. A rightward shift from 25.4 to 38.4 nm (Match–AuNP–Tat) and 37.7 nm (Scramble–AuNP–Tat) indicates surface functionalization. (I) UV–vis spectrum of ON–AuNP–Tat in water. The overlapping curves for AuNPs, Match–AuNP–Tat, and Scramble–AuNP–Tat indicate particle stability with little or no aggregation. (J) Typical TLC for ^111^In–Match–AuNP–Tat and ^111^In–Scramble–AuNP–Tat (radiochemical purity 98 and 99%, respectively).

AuNPs were functionalized with either a monofunctional layer of DTPA- or Cy3-tagged oligonucleotides (ON–AuNP) or a multifunctional layer of DTPA- or Cy3-tagged oligonucleotides plus PEG thiol (MW: 800; PEG800-SH) and Tat peptide (ON–AuNP–Tat) (Figure 1A). AuNPs were synthesized by citrate reduction of HAuCl_4_ as described previously,^40^ resulting in the formation of spherical AuNP with a mean diameter of 15.5 ± 1.9 nm and a hydrodynamic diameter in Milli-Q water of 25.4 nm [polydispersity index (PDI) 0.247] (Figure 1B,C,E). Match and Scramble sequence oligonucleotides (Table 1) with 5′-end amino modification were conjugated to the metal chelator DTPA using NHS/EDC chemistry for labeling with ^111^In or with the fluorophore Cy3 for confocal microscopy. DTPA conjugation was confirmed by radiolabeling with ^111^In, and Cy3-conjugation was assessed by UV–vis spectrometry (Figure 1D and S1). Based on the method by Rosi *et al.*, AuNPs were functionalized with 3′-end thiol-labeled oligonucleotides (ON–AuNP) by overnight incubation, salt-aging, and purification.^26^ DLS analysis and UV–vis spectrophotometry demonstrated that functionalized AuNPs did not aggregate and were monodisperse: the PDI of Match–AuNP and Scramble–AuNP was 0.23 and 0.22, respectively (Figure 1E,F). The hydrodynamic diameter of the constructs increased from 25.4 nm for unmodified AuNPs to 33.3 nm when decorated with either DTPA–Match (hereafter referred to as Match) or DTPA–Scramble (hereafter referred to as Scramble) oligonucleotides, which is consistent with an increase in size due to oligonucleotide conjugation. Labeling of Match and Scramble with ^111^In prior to AuNP functionalization allowed determination of the average number of oligonucleotides per AuNP (Match–AuNP, 76; Scramble–AuNP, 67), giving an oligonucleotide radiolabeling efficiency of 33–38%. Several mixing strategies and concentrations were tested for ON–AuNP–Tat synthesis. Direct mixing of oligonucleotides and Tat with AuNPs resulted in aggregation after salt-aging (data not shown). To minimize this undesirable aggregation, hydrophilic PEG800-SH was incorporated into the constructs. It was found that an AuNP/PEG800-SH mixing ratio of 1:3000 was sufficient to avoid aggregation when combined with AuNP/ON and AuNP/Tat ratio of 1:100 after addition of 0.3 M NaCl. DLS demonstrated that constructs were monodisperse in Milli-Q water with a PDI value of 0.33 and 0.26 for Match–AuNP–Tat and Scramble–AuNP–Tat, respectively (Figure 1H). UV–vis spectrophotometry indicated that there was no significant aggregation (Figure 1I). The hydrodynamic diameter after functionalization increased from 25.4 nm (AuNP) to 38.4 nm (Match–AuNP–Tat) and 37.7 nm (Scramble–AuNP–Tat). The total number of oligonucleotides per AuNP was 14 for Match–AuNP–Tat and 16 for Scramble–AuNP–Tat, corresponding to labeling efficiencies of 14 and 16% as determined by labeling Match and Scramble with ^111^In prior to AuNP functionalization. ON–AuNP and ON–AuNP–Tat were labeled with ^111^In and purified by centrifugation prior to each experiment. The maximum molar activity for ON–AuNP and ON–AuNP–Tat was 27 and 18 MBq/nmol oligonucleotide, respectively. The radiochemical purity after centrifugation was consistently between 90 and 95% (Figure 1G,J).

**Table 1:** Oligonucleotide Sequences and Modifications

oligonucleotide	sequence 5′–3′	modification	5′-end	3′-end
Match	CAGUUAGGGUUAG	2′OMeRNA	amine(AmC6)	thiol
Scramble	GCAGUGUGAUGAU	2′OMeRNA	amine(AmC6)	thiol

### Uptake and Trafficking of hTR-Targeting AuNP Radiopharmaceuticals

**Figure 2:**
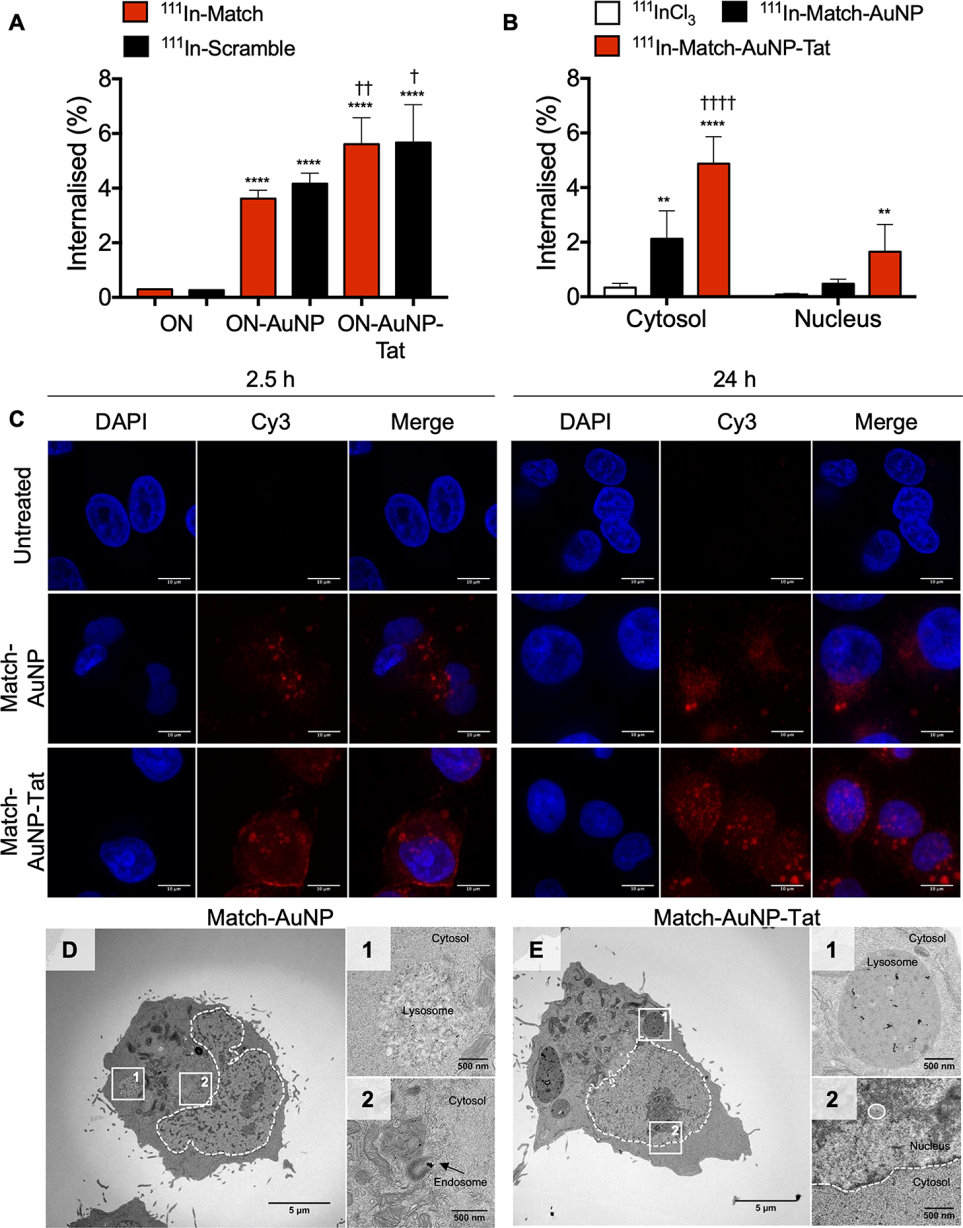
Subcellular distribution of hTR-targeting AuNPs. (A) Internalization of ON–AuNP and ON–AuNP–Tat. * = significant difference compared to free oligonucleotides (ON); † = significant difference compared to ON–AuNP (one-way ANOVA, multiple comparison, each performed in three independent experiments). (B) Intracellular distribution (fractionation assays) of AuNPs, ^111^In–Match–AuNP, and ^111^In–Match–AuNP–Tat. * = significant difference compared to ^111^InCl_3_; † = significant difference compared to ^111^In–Match–AuNP. * or †, *p* < 0.05; ** or ††, *p* < 0.01: *** or †††, *p* < 0.001; **** or ††††, *p* < 0.0001. Two-way ANOVA, multiple comparison, n = 3 replicates, four independent experiments. (C) Subcellular distribution of AuNP constructs in MDA-MB-435 cells after incubation for 2.5 or 24 h. The cells were incubated with medium only, Cy3–Match–AuNP (500 nM), or Cy3–Match–AuNP–Tat (500 nM). Images were acquired using a 63×/1.4 objective lens with an additional 4× digital zoom. Nuclei were stained with DAPI. The scale bar is 10 μm. (D) Whole-cell TEM image of Match–AuNP-treated cells. (D-1) Lysosome-like vesicle with Match–AuNP. (D-2) Endosomal vesicles with Match–AuNP. (E) Whole-cell image of Match–AuNP–Tat-treated cells. (E-1) Large lysosomal vesicle with Match–AuNP–Tat. (E-2) Nucleus with Match–AuNP–Tat.

To assess the cellular uptake of AuNP constructs, a 24 h internalization assay was performed in telomerase-positive MDA-MB-435 cells, and the uptake was measured and calculated as a percentage of the total amount of the added radioactivity (Figure 2A). The uptake of oligonucleotides was markedly improved by linking them to AuNPs: ^111^In–Match versus ^111^In–Match–AuNP, 0.30 ± 0.01 versus 3.62 ± 0.20% (*p* < 0.005); ^111^In–Scramble versus ^111^In–Scramble–AuNP, 0.26 ± 0.02 versus 4.17 ± 0.39% (*p* < 0.0001). The addition of Tat and PEG800-SH to the nanocomplex further improved cellular internalization: ^111^In–Match–AuNP versus ^111^In–Match–AuNP–Tat, 3.62 ± 0.20 versus 5.61 ± 0.969% (*p* = 0.0005); ^111^In–Scramble–AuNP versus ^111^In–Scramble–AuNP–Tat, 4.17 ± 0.39 versus 5.67 ± 1.34% (*p* = 0.0095). To further investigate the subcellular distribution of internalized AuNP constructs, fractionation assays were performed in MDA-MB-435 cells (Figure 2B). ^111^In–Match–AuNP–Tat was taken up in the cytosol to a significantly greater extent than ^111^In–Match–AuNP (4.88 ± 0.99 versus 2.13 ± 1.03%; *p* < 0.0001), and both were taken up more than ^111^InCl_3_ (0.34 ± 0.15%; *p* < 0.0001 and *p* = 0.0024 for comparison to ^111^In–Match–AuNP–Tat and ^111^In–Match–AuNP, respectively). The nuclear uptake of ^111^In–Match–AuNP–Tat (1.65 ± 1.00%) was also significantly greater than that of ^111^InCl_3_ (0.08 ± 0.044%, *p* = 0.0071). However, the difference observed in the nuclear uptake of ^111^In–Match–AuNP–Tat and ^111^In–Match–AuNP was not statistically significant (1.65 ± 1.00 versus 0.48 ± 0.17%, *p* = 0.06).

Cy3-labeled oligonucleotides were used to image AuNP intracellular localization using confocal microscopy. MDA-MB-435 cells were treated with medium only, Cy3–Match–AuNP or Cy3–Match–AuNP–Tat for 2.5 or 24 h (Figure 2C). After exposure to Cy3–Match–AuNP for 2.5 h, several intracellular perinuclear Cy3 foci were observed, confirming cellular uptake. A more intense Cy3 signal was seen for Match–AuNP–Tat, in particular at the cell membrane with some foci of fluorescence also observed in the perinuclear region. After incubation for 24 h, the Cy3 signal in Match–AuNP-treated cells was predominantly localized to large cytoplasmic foci. Some perinuclear foci were noted, but no nuclear fluorescence was observed. In contrast, in Match–AuNP–Tat-treated cells, there was a marked Cy3 signal in the perinuclear region at 2.5 h of treatment, and by 24 h, intranuclear foci were clearly observed.

To investigate the subcellular distribution with greater resolution, AuNPs were visualized in cells exposed for 24 h to Match–AuNP and Match–AuNP–Tat using TEM (Figure 2D,E). Figure 2D shows a representative TEM image demonstrating the subcellular distribution of Match–AuNP. Black dots (AuNPs) were present in endosomal vesicles in the cytosol, in agreement with the fluorescence images. Figure 2D-1 shows a large lysosome-like vesicle that contains several AuNPs. Figure 2D-2 highlights a smaller endosomal vesicle with clustered AuNPs. No particles were observed in the nucleus. In a representative image of a cell treated with Match–AuNP–Tat (Figure 2E), most particles were located in lysosomal vesicles in the perinuclear area (Figure 2E-1), but individual particles were also observed in the nucleus (Figure 2E-2). The AuNPs predominantly appear as individual particles, indicating that intracellular Match–AuNP and Match–AuNP–Tat do not aggregate (Figure S3).

### Telomerase Activity Inhibition by hTR-Targeting AuNPs

**Figure 3:**
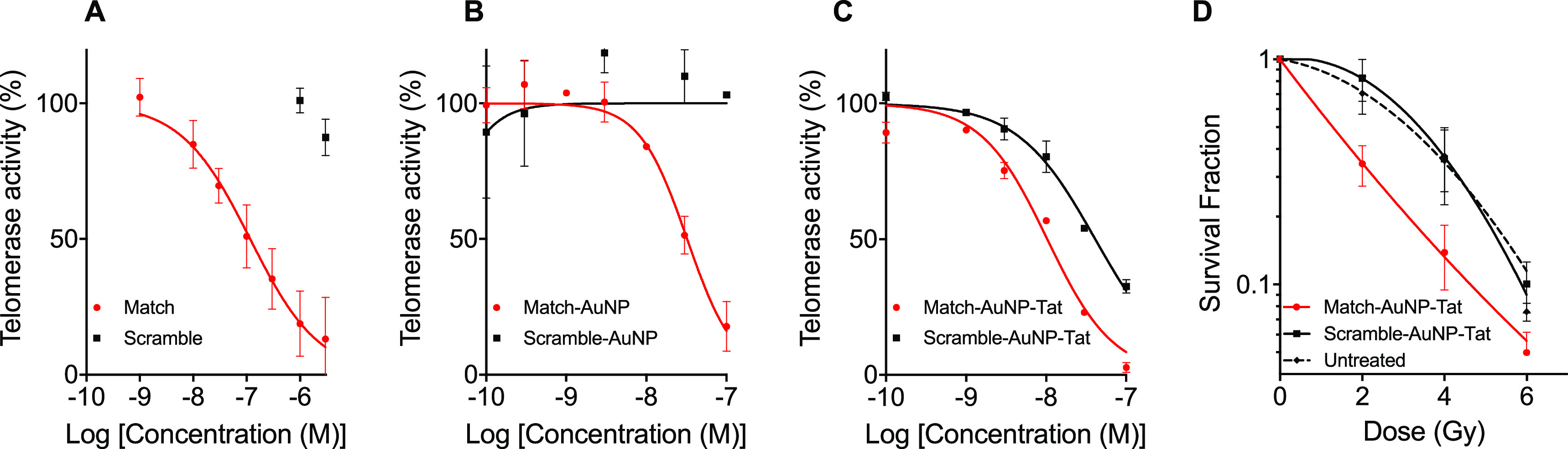
Telomerase activity inhibition by hTR-targeting AuNP constructs. (A) Dose effect of oligonucleotides on telomerase activity. The *y*-axis represents the telomerase activity signal relative to the signal of untreated cells. The *x*-axis represents the logarithm of the concentration of oligonucleotides (M). The plotted data points are the results for Match and Scramble. (B) Dose–response curve of ON–AuNP concentration versus telomerase activity. (C) Dose–response curve of ON–AuNP–Tat concentration versus telomerase activity (*n* = 3 independent experiments). (D) Effect of ON–AuNP–Tat on radiosensitivity. ON–AuNP–Tat (210 nM) was added to MDA-MB-435 cells for 24 h prior to exposure to external IR and plated for clonogenic survival analysis. Curves were plotted using the linear-quadratic model. *n* = 4, three repeats per experiment. Data points represent averages ± SEM.

The ability of AuNPs to inhibit telomerase activity was measured with a gold standard cell-free PCR-based technique, the telomeric repeat amplification protocol (TRAP) assay (Figure S2A). Match, but not Scramble, oligonucleotides inhibited telomerase in telomerase-expressing MDA-MB-435 (log IC_50_ −6.93 ± 0.072) (Figure 3A), consistent with previous results.^21^ The effect of ON–AuNPs on telomerase activity was also tested using TRAP assays (Figure 3B). Match–AuNP inhibited the telomerase activity in a dose-dependent manner (log IC_50_ −7.49 ± 0.04, Hill slope −1.46 ± 0.19). Scramble–AuNP did not significantly alter the telomerase activity over the concentration range of 0.1–100 nm. Successful amplification of a pre-elongated primer, TSR8, demonstrated that the construct did not inhibit the telomerase-independent downstream steps of the TRAP assay (Figure S2B).

TRAP assays were also conducted following exposure of cells to ON–AuNP–Tat constructs (Figure 3C). Match–AuNP–Tat inhibited the telomerase activity in a dose-dependent manner (log IC_50_ −7.99 ± 0.05, Hill slope −1.05 ± 0.27). Scramble–AuNP–Tat also demonstrated a dose-dependent relationship (log IC_50_ −7.39 ± 0.03, Hill slope −0.90 ± 0.06), although log IC_50_ was significantly higher than for Match–AuNP–Tat (*p* < 0.0001). This result raised the possibility that the Scramble–AuNP–Tat construct itself interferes with the quantification technique, rather than specifically inhibiting the telomerase activity. Consistent with this, a significant reduction in the TRAP assay signal was observed when the Tat-functionalized AuNP was added to TSR8, indicating a telomerase-independent experimental artifact (Figure S2C).

### Match–AuNP–Tat Sensitizes Cells to External Beam Radiation

Telomerase inhibition has been reported to radiosensitize cells to IR,^16,19,41^ and AuNPs also demonstrate this property.^42^ To investigate whether AuNPs and AuNP-mediated delivery of hTR-inhibiting oligonucleotides were able to sensitize cells to externally delivered IR, MDA-MB-435 cells were treated with non-radiolabeled ON–AuNP–Tat (210 nM), exposed to external beam IR (0-6 Gy), and then processed for clonogenic survival assays (Figure 3D). The MID of Match–AuNP–Tat (2.02 ± 0.26) was significantly lower than for the untreated (control) (3.23 ± 0.56, *p* = 0.022) and Scramble–AuNP–Tat-treated samples (3.48 ± 0.66, *p* = 0.008). This resulted in an SER of 1.60 ± 0.12 for Match–AuNP–Tat compared to an SER of 0.93 ± 0.06 for Scramble–AuNP–Tat. The greater SER for match–AuNP–Tat is attributed to its telomerase-inhibiting properties resulting in radiosensitization.

### hTR-Targeting AuNP Radiopharmaceuticals Reduce Clonogenic Survival by Increasing Radiation-Induced DNA Double-Strand Breaks

**Figure 4:**
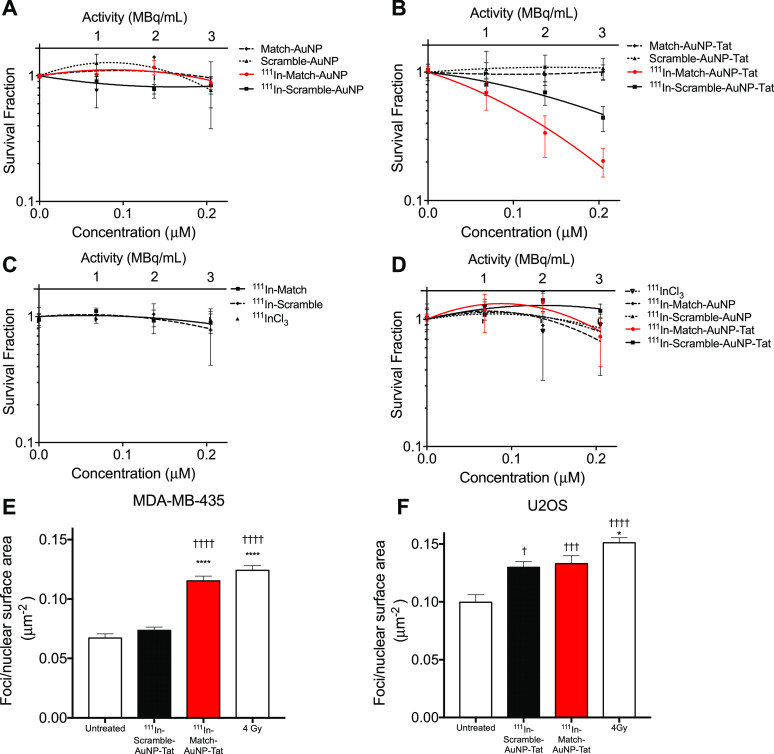
Radiobiological impact of radiolabeled hTR-targeting AuNPs. Clonogenic survival of MDA-MB-435 cells after treatment for 24 h with (A) ON–AuNP, (B) ON–AuNP–Tat, and (C) ^111^InCl_3_, ^111^In–Scramble, and ^111^In–Match. (D) Clonogenic survival of U2OS after treatment for 24 h with ^111^In–ON–AuNP and ^111^In–ON–AuNP–Tat. In (A–D), curves were plotted using the linear quadratic model; *n* = 4–6, three replicates per experiment. The *y*-axis represents the cell survival fraction. The *x*-axis represents the logarithm of the concentration of oligonucleotides (M). Data points represent the mean ± SEM. γH2AX foci after treatment of (E) MDA-MB-435 and (F) U2OS cells with ^111^In–ON–AuNP–Tat. Cells were treated with ^111^In–ON–AuNP–Tat (210 nM) or control for 24 h before fixation and staining for γH2AX. † = significant difference compared to untreated control. * = significant difference compared to ^111^In–Scramble–AuNP–Tat. † or *, *p* < 0.05; †† or **, *p* < 0.01: ††† or ***, *p* < 0.001; †††† or ****, *p* < 0.0001. Differences between samples were analyzed using one-way ANOVA, multiple comparison (*n* = 65–125 cells).

Since the ON–AuNP constructs are designed to be deployed as radiopharmaceuticals, the clonogenic survival of cells exposed to radiolabeled ON–AuNP and ON–AuNP–Tat was evaluated using the linear quadratic model in telomerase-positive MDA-MB-435 cells and telomerase-negative U2OS cells (Figure 4A–D). ^111^In-labeled ON–AuNP (Match and Scramble) constructs had no significant impact on clonogenic survival of MDA-MB-435 cells (Figure 4A) (*p* = 0.23). In contrast, ^111^In–Match–AuNP–Tat significantly reduced clonogenic survival (SF at 210 nm ^111^In–Match–AuNP–Tat: 0.20 ± 0.11). ^111^In–Match–AuNP–Tat showed a greater cell killing effect than ^111^In–Scramble–AuNP–Tat (MID: 0.11 ± 0.02 versus 0.15 ± 0.02, *p* = 0.03, one-way ANOVA, multiple comparison), indicating a sequence-dependent effect of ^111^In–ON–AuNP–Tat (Figure 4B). Furthermore, ^111^In–Match–AuNP–Tat had a significant impact on clonogenic survival compared to non-radiolabeled Tat-modified AuNP control: Match–AuNP–Tat (*p* = 0.006), Scramble–AuNP–Tat (*p* = 0.002). ^111^In–Scramble–AuNP–Tat showed a similar, although less pronounced, difference compared to controls (*p* = 0.0237 for Match–AuNP–Tat, *p* = 0.0037 for Scramble–AuNP–Tat). Unconjugated ^111^InCl_3_, ^111^In-Match, and ^111^In-Scramble did not significantly alter clonogenic survival (Figure 4C). A control experiment in U2OS cells, which lack telomerase activity, demonstrated a modest reduction in clonogenic survival following treatment with ^111^In–Match–AuNP–Tat and ^111^In–Scramble–AuNP–Tat at the highest concentration (SF 0.73 ± 0.53 and 0.73 ± 0.37, respectively). This reduction did not differ significantly from control treatments with ^111^InCl_3_, ^111^In–Match–AuNP, and ^111^In–Scramble–AuNP (Figure 4D), indicating a modest telomerase-independent effect.

To elucidate whether the observed radiotoxic effect of AuNP- and Tat-mediated internalization of ^111^In is caused by an increase in DNA damage, MDA-MB-435 and U2OS cells were treated with radiolabeled AuNP constructs. Following incubation for 24 h, the cells were exposed to the anti-γH2AX antibody, as γH2AX is a well-validated marker of DNA double-strand breaks (DSB).^43^ Cells exposed to 4 Gy external beam IR were used as a positive control. Strong induction of γH2AX was noted in MDA-MB-435 cells following 4 Gy of external beam IR (foci/NSA was 0.12 ± 0.04 μm^–2^) (Figure 4E). ^111^In–Match–AuNP–Tat also caused a highly significant upregulation of γH2AX foci/NSA in comparison to an untreated control (0.12 ± 0.04 versus 0.07 ± 0.03 μm^–2^; *p* < 0.0001) and to ^111^In–Scramble–AuNP–Tat (0.12 ± 0.04 versus 0.07 ± 0.03 μm^–2^; *p* < 0.0001). There was no difference between untreated control and Scramble–AuNP–Tat. Furthermore, the number of foci induced by ^111^In–Match–AuNP–Tat was similar to that induced by IR (4 Gy)-treated positive control cells (*p* = 0.303). The telomerase-dependency of this effect was assessed using the telomerase-negative cell line U20S (Figure 4F).

As with MDA-MB-435, robust induction of foci was observed after treatment with 4 Gy IR compared to the untreated control (*p* < 0.0001). ^111^In–Scramble–AuNP–Tat caused a modest induction of γH2AX foci in U2OS cells as did ^111^In–Match–AuNP–Tat in this cell line. Both AuNP constructs had a significantly higher number of foci/NSA than the untreated control (^111^In–Scramble–AuNP–Tat: *p* = 0.0017; ^111^In–Match–AuNP–Tat: *p* = 0.0004) but did not show a difference to each other (*p* = 0.9818). These results indicate that the ability of ^111^In–Match–AuNPs to induce DSBs is mediated by telomerase activity inhibition.

### Biodistribution of ^111^In–ON–AuNP–Tat

A pilot study of the *in vivo* biodistribution of ^111^In–Match–AuNP–Tat and ^111^In–Scramble–AuNP–Tat was performed in a murine MDA-MB-435 xenograft model. Employing a combination of SPECT imaging and *ex vivo* radioactivity quantification, radiolabeled AuNPs demonstrated high liver and spleen sequestration along with low tumor accumulation, 0.7% ID/g (Figure S4).

## Discussion

Since the discovery of telomerase, strategies aiming to inhibit this enzyme have been of great interest to the cancer research community. Several studies have reported that optimal tumor control has been achieved when telomerase inhibition is combined with either external beam radiation or chemotherapy.^20,44−46^ It was therefore hypothesized that the combination of telomerase inhibition plus radionuclide therapy would be beneficial in the treatment of telomerase-positive malignancies. This study is the first to report that ^111^In-labeled anti-hTR oligonucleotide-functionalized AuNPs have a specific anticancer therapeutic potential. Both the ON–AuNP and ON–AuNP–Tat constructs delivered their payload into cells with a roughly 10–20 times higher efficiency than free oligonucleotides, strongly improving the uptake kinetics that were reported previously.^21^ The extent of internalization of AuNPs was comparable to previous reports by us and others.^30,47,48^ Match–AuNP–Tat was internalized significantly more than Match–AuNP, and there was a marked difference in the fate of these two constructs after internalization. Match–AuNP were concentrated in small foci around the cell membrane and in the cytoplasm and were not observed outside endosomal vesicles in TEM images, which is in agreement with earlier research by Brandenberger *et al.* (2010) who showed that AuNPs remained within vesicles in the perinuclear space.^49^

The addition of PEG800-SH and Tat to the AuNP ligand shell markedly enhanced the uptake kinetics of the particle. The internalization of Cy3–Match–AuNP–Tat after 2.5 h resulted in the formation of intense foci, indicating clustering in endosomal vesicles, but a less intense uniform signal was also noted in the cytosol and nucleus. This was more obvious after 24 h, when high-density Cy3 focal spots were detected in the perinuclear area and a more diffuse signal through the cytosol and nucleus. This correlated with findings by TEM, where AuNP dots were found in endosomal vesicles, as well as in the nucleus and cytosol. A similar phenomenon was observed by de la Fuente and Berry (2005), who used Tat for the delivery of 2.4–8.2 nm AuNPs.^32^ Consistent with this, Nativo *et al.* (2008) found that AuNPs loaded with PEG-SH, Tat, and two other NLS exhibited perinuclear localization after internalization.^50^ These investigators suggested that the accumulation of Tat-functionalized AuNPs in unusual perinuclear membranous structures was the result of disruption of endosomal membranes. Although perinuclear accumulation was also detected in the current study, TEM revealed no evidence of endosomal disruption. While a favorable intracellular distribution of ^111^In-ON–AuNP-Tat was observed in *in vitro* analyses, limited tumor accumulation *in vivo* was noted. These findings are in agreement with a recent meta-analysis by Wilhelm *et al.* and highlight the need for further optimization of the delivery system through, for example, addition of a tumor-specific ligand.^51^

In line with the data reported previously, it was shown that telomerase activity can be inhibited by anti-hTR 2′OMeRNA oligonucleotides in a concentration-dependent manner.^21,52^ Using the TRAP assay, addition of Match to AuNP resulted in inhibition of telomerase activity (log IC_50_ −7.99 ± 0.05), indicating that oligonucleotide conjugation to AuNP did not hamper the anti-telomerase effect of Match. However, although Scramble–AuNP did not exert an effect on telomerase enzymatic activity, Scramble–AuNP–Tat did induce dose-dependent telomerase inhibition (log IC_50_ −7.39 ± 0.03). This apparently inconsistent result may be due to the presence of Tat, which is highly positively charged and which disrupted the PCR amplification stages of the TRAP assay. The interference of this assay is a phenomenon that has been reported in the literature^53^ and makes the result of the TRAP assay hard to interpret in the presence of Tat. However, it is interesting to point out that the inhibitory effect of Match–AuNP–Tat is significantly higher than that of Scramble–AuNP–Tat. This suggests that Match–AuNP–Tat may have a specific effect on telomerase, which is detectable even when the sensitivity of the assay is impaired due to the interference by Tat.

Previously, researchers have demonstrated that hTR-binding oligonucleotides can cause sensitization to external IR.^14−20^ To investigate whether there was a telomerase-specific radiosensitizing effect when oligonucleotides were conjugated to AuNPs, the cells were incubated with non-radiolabeled Match–AuNP–Tat constructs and exposed to increasing doses of externally delivered IR. The result of this experiment showed a highly radiosensitizing effect with an SER for Match–AuNP–Tat of 1.6, which is in line with the results reported by Wu *et al.* (2017).^19^ These authors showed that treatment with imetelstat prior to irradiation of Kyse410 and Kyse520 cells led to SERs of 1.9 and 1.6, respectively. However, although Wu *et al.* treated cells for 40 days prior to irradiation, our results suggest that a 24 h treatment is sufficient to elicit radiosensitization. This indicates that telomere shortening is not the sole mechanism of action of hTR inhibition-induced radiosensitization.

^111^In–Match–AuNP–Tat had a significant, dose-dependent effect on the clonogenic survival of MDA-MB-435 cells. ^111^In–Scramble–AuNP–Tat also led to a reduction in clonogenicity, although this construct was significantly less effective. The effect of the latter compound is likely the result of cellular internalization of radiotoxic ^111^In.^54^ In this study, a correlation is shown between the cytosolic and nuclear uptake of particles and their capacity to reduce clonogenic survival of the telomerase-positive cell line MDA-MB-435. Both Match–AuNP and Match–AuNP–Tat inhibit the telomerase activity in a cell-free system, but only ^111^In-labeled Match–AuNP–Tat has a significant and telomerase-specific effect on clonogenic survival. There is a discrepancy in the subcellular distribution of both constructs, which is hypothesized to be the result of the addition of Tat. For the constructs to inhibit hTR, they must reach the nucleus. Furthermore, the effect of ^111^In is most profound within a range of about 11 nm from DNA.^55^ Since Match–AuNP–Tat is present in the nucleus in greater amount than Match–AuNP, this may explain the greater reduction in clonogenic survival with the Tat-modified construct. The difference in effect between ^111^In–Match–AuNP–Tat and ^111^In–Scramble–AuNP–Tat can be explained by the difference in oligonucleotide sequence in the ligand shell of the two constructs and is consistent with effective targeting of telomerase by ^111^In–Match–AuNP–Tat but not ^111^In–Scramble–AuNP–Tat. Crucially, there is no difference in effect between these two constructs in cells that lack telomerase expression (Figure 4F). Furthermore, these constructs have a minimal effect on clonogenic survival when not radiolabeled, which is keeping with previous findings.^56−58^ Chen *et al.* reported that a treatment time of 2 weeks with a 2′OMe anti-hTR oligonucleotide was enough to exert a telomerase-specific radiosensitizing effect.^56^ Interestingly, this time would be too short to induce critical telomere shortening, implicating a non-canonical effect of hTR inhibition. This correlates with the direct induction of DNA damage observed in the current study which, it is proposed, is the underlying cause of the reduction in clonogenic survival. Non-canonical properties of hTR have been described and may account for these observed effects. hTR has been shown to protect against oxidative stress and apoptosis,^59,60^ as well as to stimulate DNA-dependent kinase (DNA-PKcs) to phosphorylate hnRNPA1, a protein critical for capping telomeres.^61,62^ Impairment of these functions may result in increased DNA damage when combined with radiation.

When taken together, the data showing sequence-specific and radiolabel-dependent effects lead to the conclusion that short-term hTR inhibition results in sensitization to the radiotoxic effects of Auger electrons and external irradiation. In agreement with this, we observed strong upregulation of γH2AX foci after treatment with ^111^In–Match–AuNP–Tat but not after treatment with ^111^In–Scramble–AuNP–Tat in telomerase-positive cells. Furthermore, there was a modest upregulation of γH2AX foci following exposure of telomerase-negative cells to ^111^In–Match–AuNP–Tat and ^111^In–Scramble–AuNP–Tat, but this was sequence-independent, and likely the result of modest accumulation of intranuclear ^111^In. These results suggest that ^111^In–Match–AuNP–Tat either causes more DNA damage than ^111^In–Scramble–AuNP–Tat or inhibits the repair of such damage in telomerase-positive cells. The results shown here are in line with our previous work, which demonstrated that ^111^In-labeled anti-hTR oligonucleotides have a telomerase-specific anti-proliferative and cytotoxic effect *via* the induction of DNA damage in three telomerase-positive cell lines.^21^ The current study builds on this, demonstrating that AuNPs can function as efficient cellular delivery vehicles for radiolabeled oligonucleotides, eliminating the need for non-clinically relevant transfectants.

## Conclusions

In this paper, progress toward effective cellular delivery of ^111^In-labeled hTR-targeting oligonucleotides is reported. AuNP constructs functionalized with a multivalent ligand shell of PEG800-SH, Tat and ^111^In-labeled oligonucleotides exhibited a superior subcellular distribution profile in comparison to AuNP constructs with a monovalent ligand shell of ^111^In-labeled oligonucleotides only. Treatment with ^111^In–Match–AuNP–Tat led to a sequence- and telomerase-dependent DNA damage-induced reduction in clonogenic survival of malignant cells, underlining its potential as a specific anticancer agent. Overall, this work supports the concept that nucleic acid-based anticancer radiopharmaceuticals represent a promising strategy for targeting tumors.

## Supporting Information Available

Conjugation of Cy3, telomerase assay controls, in situ Match–AuNP and Match–AuNP–Tat TEM images, and SPECT imaging and ex vivo biodistribution of ^111^In–ON–AuNP–Tat. (PDF)
