# Supplementary material for: Oligonucleotide-Functionalized Gold Nanoparticles for Synchronous Telomerase Inhibition, Radiosensitization, and Delivery of Theranostic Radionuclides
Source: Mol Pharm. 2021 Aug 27;18(10):3820–31. doi: 10.1021/acs.molpharmaceut.1c00442 (PMC8493550; doi:10.1021/acs.molpharmaceut.1c00442)
Supplement: Supplementary file 1 — mp1c00442_si_001.pdf [file mp1c00442_si_001.pdf]

Supporting Information

Oligonucleotide-functionalized gold nanoparticles for synchronous telomerase inhibition, radiosensitization and delivery of theranostic radionuclides

Bas M. Bavelaar, Lei Song, Mark R. Jackson, Sarah Able, Ole Tietz, Irini Skaripa-Koukelli, Philip A. Waghorn, Martin R. Gill, Robert C. Carlisle, Madalena Tarsounas, Katherine A. Vallis

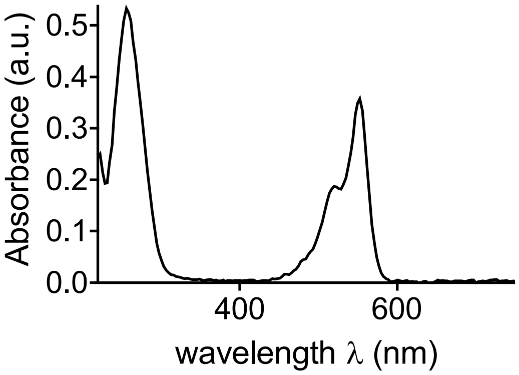

Fig. S1. Conjugation of Cy3 to Match oligonucleotides. UV-Vis spectrum of Cy3-labeled Match oligonucleotides. Two distinct peaks are visible at λ = 260 nm (oligonucleotides) and at λ = 548 nm (Cy3), indicating successful conjugation.

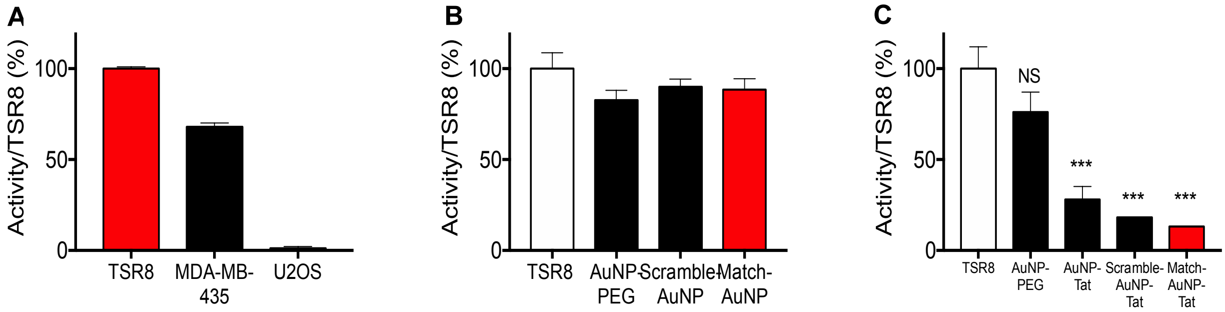

Fig. S2. (A) Telomerase activity in MDA-MB-435 and U2OS cell lines. The y-axis represents the telomerase activity relative to the signal of 0.2 amole of TSR8 telomerase-independent pre-elongated primer. (n = 2). (B) Influence of ON-AuNP constructs on TSR8 signal. (C) Influence of ON-AuNP-Tat on TSR8 signal. NS = not significant, *** = $p < 0.001$.

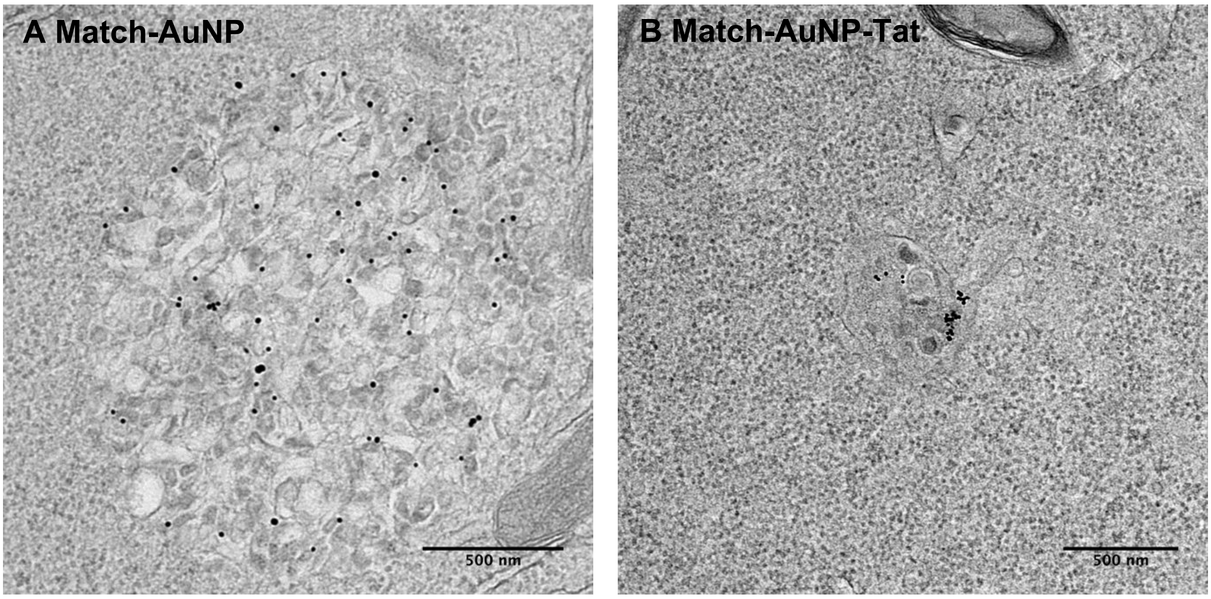

Fig. S3. (A) Match-AuNP in a large lysosome. Most Match-AuNP are visible as individual particles, although some clusters can be found. (B) Match-AuNP-Tat in an endosome. Match-AuNP-Tat appear mostly as individual particles, but some are clustered

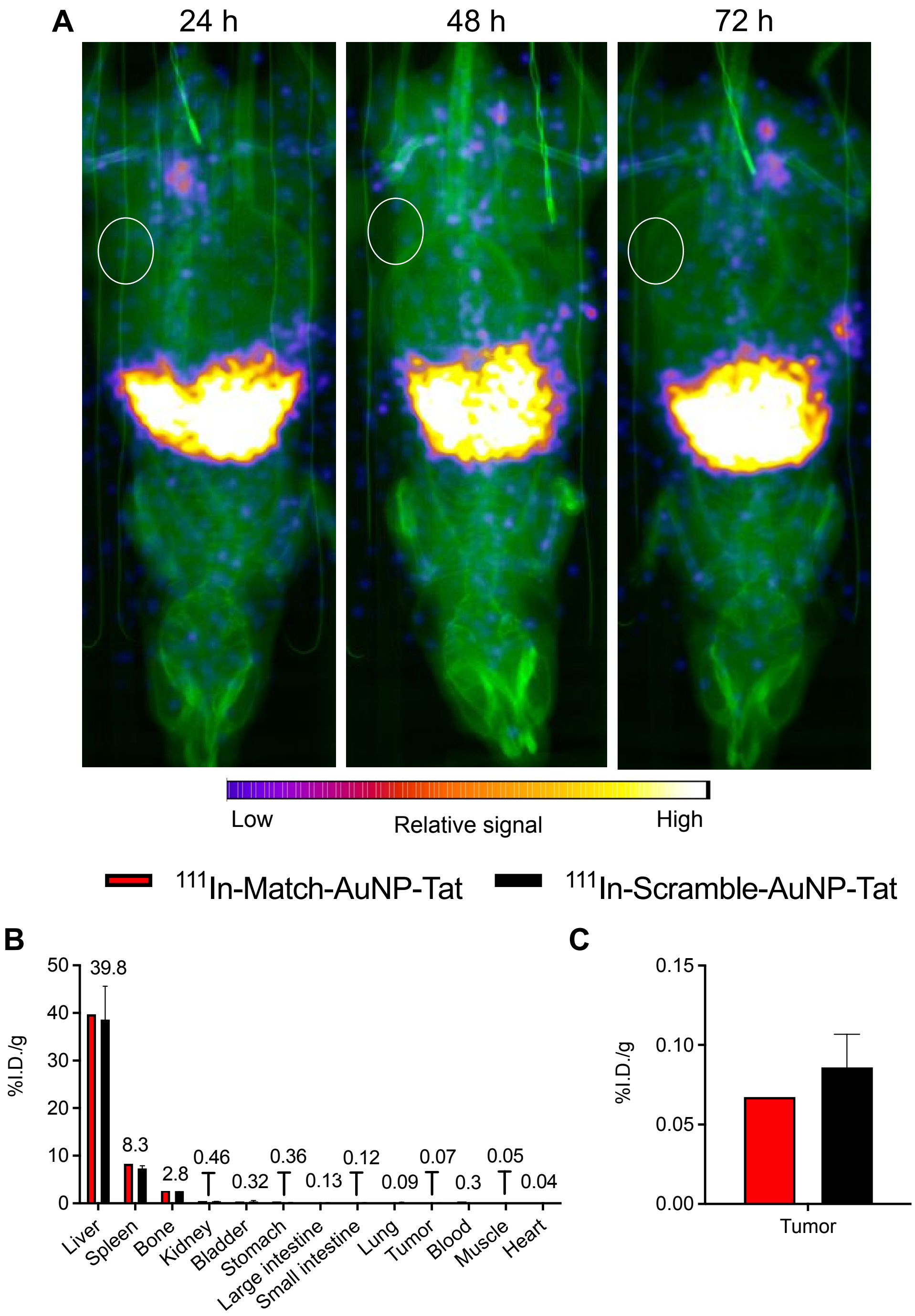

Fig. S4. SPECT imaging and ex vivo biodistribution of ^{111}In-ON-AuNP-Tat. (A) Whole-body SPECT showing biodistribution of ^{111}In-Match-AuNP-Tat in MDA-MB-435 xenograft-bearing mice. Images of the same mouse acquired at 24, 48 and 72 h p.i. are shown and are representative of a group of 3 mice. Ex vivo analysis was performed 72 h post-injection. The amount of radioactivity in selected tissues/organs (B) and tumours (C) was measured and data expressed as %I.D./g. Error bars represent mean ± SD (n=3/group). Numbers above bars indicate the average for ^{111}In-Match-AuNP-Tat. A Two-Way ANOVA was performed: there was no statistically significant difference between ^{111}In-Match-AuNP-Tat and ^{111}In-Scramble-AuNP-Tat uptake in organs or tumour. %I.D./g: Percentage of the injected dose per gram of tissue.
